# A Population-based Study on Lymph Node Retrieval in Patients with Esophageal Cancer: Results from the Dutch Upper Gastrointestinal Cancer Audit

**DOI:** 10.1245/s10434-018-6396-7

**Published:** 2018-03-09

**Authors:** L. R. van der Werf, J. L. Dikken, M. I. van Berge Henegouwen, V. E. P. P. Lemmens, G. A. P. Nieuwenhuijzen, B. P. L. Wijnhoven, the Dutch Upper GI Cancer Audit group, K. Bosscha, N. C. T. van Grieken, H. H. Hartgrink, R. van Hillegersberg, V. E. P. P. Lemmens, J. T. Plukker, C. Rosman, J. W. van Sandick, P. D. Siersema, G. Tetteroo, P. M. J. F. Veldhuis, F. E. M. Voncken

**Affiliations:** 1 Department of Surgery, Erasmus University Medical Centre, Rotterdam, The Netherlands; 2 Department of Surgery, Leiden University Medical Centre, Leiden, The Netherlands; 3 Department of Surgery, Amsterdam Medical Centre, Amsterdam, The Netherlands; 4 Department of Epidemiology, Erasmus University Medical Centre, Rotterdam, The Netherlands; 5 Department of Surgery, Catherina Hospital, Eindhoven, The Netherlands

## Abstract

**Background:**

For esophageal cancer, the number of retrieved lymph nodes (LNs) is often used as a quality indicator. The aim of this study is to analyze the number of retrieved LNs in The Netherlands, assess factors associated with LN yield, and explore the association with short-term outcomes. This is a population-based study on lymph node retrieval in patients with esophageal cancer, presenting results from the Dutch Upper Gastrointestinal Cancer Audit.

**Study Design:**

For this retrospective national cohort study, patients with esophageal carcinoma who underwent esophagectomy between 2011 and 2016 were included. The primary outcome was the number of retrieved LNs. Univariable and multivariable regression analyses were used to test for association with ≥ 15 LNs.

**Patients and Results:**

3970 patients were included. Between 2011 and 2016, the median number of LNs increased from 15 to 20. Factors independently associated with ≥ 15 LNs were: 0–10 kg preoperative weight loss (versus: unknown weight loss, odds ratio [95% confidence interval]: 0.71 [0.57–0.88]), Charlson score 0 (versus: Charlson score 2: 0.76 [0.63–0.92]), cN2 category (reference: cN0, 1.32 [1.05–1.65]), no neoadjuvant therapy and neoadjuvant chemotherapy (reference: neoadjuvant chemoradiotherapy, 1.73 [1.29–2.32] and 2.15 [1.54–3.01]), minimally invasive transthoracic (reference: open transthoracic, 1.46 [1.15–1.85]), open transthoracic (versus open and minimally invasive transhiatal, 0.29 [0.23–0.36] and 0.43 [0.32–0.59]), hospital volume of 26–50 or > 50 resections/year (reference: 0–25, 1.94 [1.55–2.42] and 3.01 [2.36–3.83]), and year of surgery [reference: 2011, odds ratios (ORs) 1.48, 1.53, 2.28, 2.44, 2.54]. There was no association of ≥ 15 LNs with short-term outcomes.

**Conclusions:**

The number of LNs retrieved increased between 2011 and 2016. Weight loss, Charlson score, cN category, neoadjuvant therapy, surgical approach, year of resection, and hospital volume were all associated with increased LN yield. Retrieval of ≥ 15 LNs was not associated with increased postoperative morbidity/mortality.

**Electronic supplementary material:**

The online version of this article (10.1245/s10434-018-6396-7) contains supplementary material, which is available to authorized users.

Since the relationship between the number of retrieved LNs and survival was shown, the number of retrieved lymph nodes (LNs) has often been used as a quality indicator for esophageal cancer surgery.^1^^–^5

In 2013, the total number of retrieved LNs was introduced as one of the quality indicators in the Dutch Upper Gastrointestinal Cancer Audit (DUCA).^6^ This nationwide audit aims to provide insight into the quality of delivered care by reporting reliable and benchmarked information on process and outcome parameters, defined as “quality indicators.” The 7th edition of the Union for International Cancer Control (UICC)/American Joint Committee on Cancer (AJCC) classification recommended removal of at least 15 LNs for reliable staging of gastric cancer.^7^ Hence, the number of 15 nodes was introduced as a quality indicator for esophageal cancer.

It is unclear whether introduction of this indicator resulted in higher LN yield. Furthermore, it is unknown which factors are associated with the number of LNs retrieved and whether higher LN yield is associated with higher postoperative morbidity or mortality.

The aim of this study is to evaluate trends in the number of retrieved lymph nodes and the proportion of patients with ≥ 15 LNs in the resection specimen. The second aim is to identify patient, tumor, and treatment factors associated with the number of retrieved LNs, LN yield, and thirdly, to evaluate whether higher LN yield is associated with increased morbidity and/or 30-day/in-hospital mortality.

## Methods

### Study Design

Data were retrieved from the DUCA. This surgical audit was initiated in 2011 and is part of the Dutch Institute for Clinical Auditing (DICA). All patients with esophageal or gastric cancer with intent of resection should be registered. Results on quality indicators are reported to the participating hospitals. Each year, external quality indicators are made transparent to the public, policy-makers, insurance companies, and patient federations. Validation of completeness and accuracy of data registration is performed.^6^ For this study, patient, tumor, and treatment characteristics, pathological information, and postoperative outcome (until 30 days after operation) were retrieved from the DUCA. Because patient and hospital identity are anonymous in the database, it was not possible to retrieve missing data or additional variables in retrospect.

### Patient Selection

All patients undergoing surgery for esophageal cancer with curative intention between 2011 and 2016 were included. Patients with unknown date of birth, unknown survival status at 30 days after surgery or discharge (in case of hospital stay > 30 days), or with unknown number of retrieved LNs were excluded.

Since 2010, nCRT followed by surgery has been the standard treatment according to the Dutch guideline for esophageal carcinoma (with the exception of T1N0 tumors).^8^

### Outcomes

Primary outcomes were the number of retrieved LNs (as documented by the pathologist based on examination of the resection specimen) and the percentage of patients with ≥ 15 LNs retrieved (as defined by the number of patients with at least 15 retrieved LNs relative to the total number of patients who underwent resection).

No informed consent or ethical approval was required under Dutch law.

### Statistical Analysis

To compare patient, tumor, and treatment characteristics and surgical outcomes between the groups with ≥ 15 LNs and with < 15 LNs, the *χ*^2^ test was used. To identify associated factors, univariable and multivariable logistic regression analyses were performed. Factors with *P* value < 0.10 on univariable analyses or clinically relevant were included in multivariable analyses. For all analyses, statistical significance was defined as *P* < 0.05. All analyses were performed using SPSS^®^ version 24 (IBM, Armonk, NY, USA) and R (R Studio, version 0.99.903, Inc., with package “ggplot2”).

Possible factors associated with LN yield were selected by the scientific committee of the DUCA based on literature. Consensus was reached for the factors age, preoperative weight loss, Body Mass Index (BMI), tumor location, American Society of Anesthesiologists (ASA) score, Charlson comorbidity score,^9^ clinical T-, N-, and M-category of the tumor, neoadjuvant chemo(radio)therapy, surgical approach (minimally invasive or open, and transhiatal or transthoracic), annual hospital volume, and year of surgery. For evaluation of minimally invasive approaches, stratified multivariable analysis for transhiatal and transthoracic was used. To assess the relationship between ≥ 15 LNs and surgical outcomes, yield of ≥ 15 LNs was analyzed in relation to nonradicality of the resection (resection margins not free of tumor cells), intraoperative complications, postoperative complications, and 30-day and/or in-hospital mortality. A severe complication was defined as a complication leading to hospital stay > 21 days, reintervention or death.

## Results

A total of 4076 patients who underwent esophagectomy for esophageal carcinoma were registered in the DUCA between 2011 and 2016. Some patients were excluded because date of birth was missing (*n* = 12), survival status after 30 days/at discharge was missing (*n* = 80), or the number of LNs was not documented (*n* = 14). Hence, a total of 3970 patients was included in the study analyses (Supplementary Fig. S1).

### Number of Retrieved LNs

**Fig. 1:**
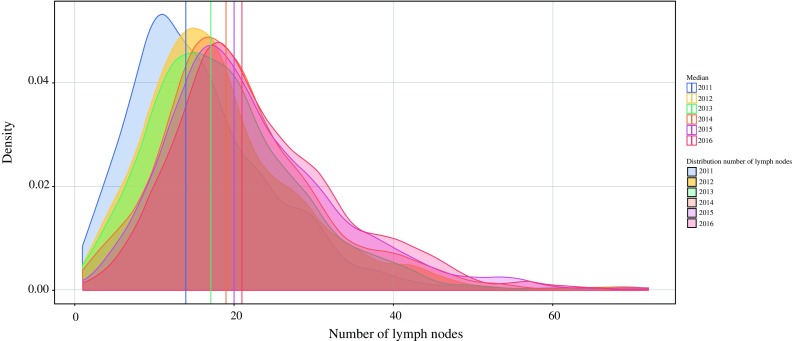
Density plot showing distribution of absolute number of LNs retrieved from 2011 to 2016

**Fig. 2:**
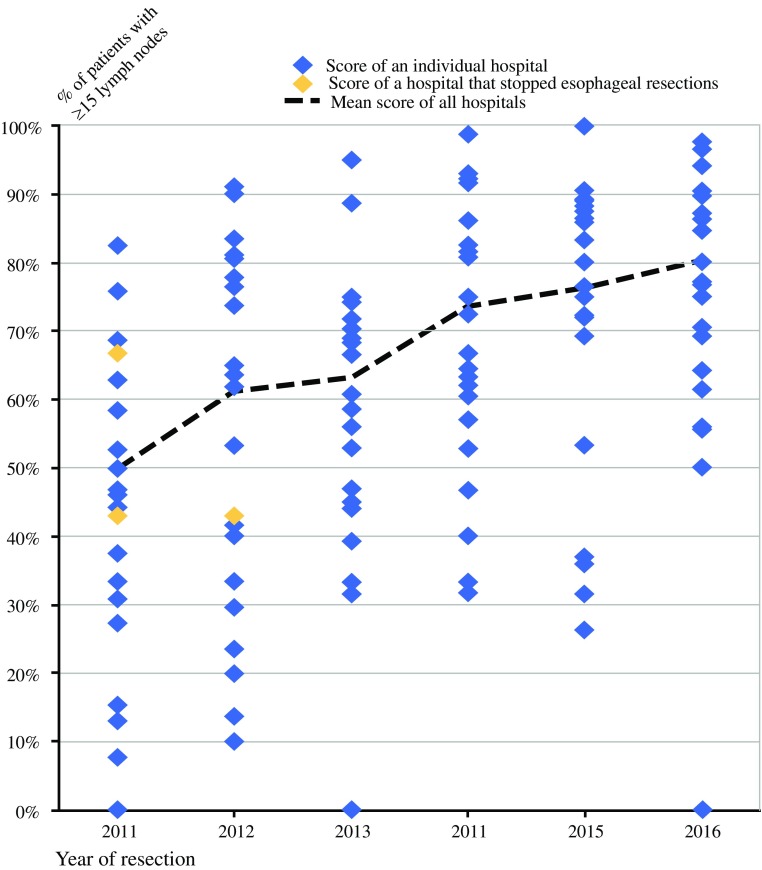
Variation in hospital score on the quality indicator “retrieval of at least 15 LNs”

The median number of retrieved LNs increased from 15 [interquartile range (IQR) 10–21] in 2011 to 20 (IQR 16–27) in 2016 (Fig. 1). Overall, the percentage of patients with ≥ 15 LNs was 69%. Among patients with ≥ 15 LNs, the median number of retrieved LNs was 22 (IQR 18–28), and in the group of patients with < 15 LNs, this number was 11 (IQR 8–13). The percentage of patients with ≥ 15 retrieved LNs increased from 51% in 2011 to 81% in 2016. In 2011, the percentage of patients with ≥ 15 retrieved LNs ranged between 0 and 77% among hospitals. In 2016, this variation between hospitals decreased (Fig. 2).

### Factors Associated with ≥ 15 LNs

Patient, tumor, treatment, and hospital characteristics are presented in Table 1. Factors associated with < 15 LNs were Charlson score 2 (reference: Charlson score 0, 0.76 [0.63–0.92]) and unknown preoperative weight loss (reference: 0–10 kg weight loss, odds ratio [95% confidence interval] 0.71 [0.57–0.88]) (Table 2).

**Table 1:** Basic characteristics of study population, including score of % of patients with ≥ 15 lymph nodes for each subgroup

Patient characteristic	Total *n* (%)	Results on the quality indicator		
		< 15 LNs	≥ 15 LNs	*P* value*
Total	3970	31%	69%	
Gender				0.83
Male	3077 (78%)	31%	69%	
Female	892 (23%)	31%	69%	
Unknown	1 (0.0%)	0%	100%	
Age (in years)				0.002
0–64	1787 (45%)	29%	71%	
65–74	1650 (42%)	31%	69%	
75 +	533 (13%)	37%	63%	
Preoperative weight loss (kg)				< 0.001
0–5	2154 (54%)	29%	71%	
6–10	835 (21%)	31%	69%	
10 +	443 (11%)	33%	67%	
Unknown	538 (14%)	38%	62%	
Body Mass Index (kg/m^2^)				0.48
< 20	257 (6.5%)	34%	66%	
20–24	1512 (38%)	31%	70%	
25–29	1522 (38%)	30%	70%	
30 +	635 (16%)	33%	67%	
Unknown	44 (1.1%)	41%	59%	
Tumor location in esophagus				< 0.001
Cervical	4 (0.1%)	50%	50%	
Proximal	40 (1.0%)	15%	85%	
Mid	486 (12%)	25%	76%	
Distal	2504 (63%)	31%	69%	
Gastroesophageal junction	936 (24%)	36%	65%	
ASA score				0.08
I–II	3070 (77%)	30%	70%	
III +	880 (22%)	33%	67%	
Unknown	20 (0.5%)	50%	50%	
Charlson score				0.002
0	1939 (49%)	29%	71%	
1	1012 (26%)	31%	69%	
2 +	1019 (26%)	35%	65%	
Clinical T-category				0.63
cT0–1	209 (5.3%)	29%	71%	
cT2	736 (19%)	33%	67%	
cT3	2731 (69%)	31%	69%	
cT4	135 (3.4%)	29%	71%	
Unknown	159 (4.0%)	31%	69%	
Clinical N-category				0.001
cN0	1407 (35%)	33%	67%	
cN1	1591 (40%)	31%	69%	
cN2	716 (18%)	26%	74%	
cN3	100 (2.5%)	25%	75%	
cN+ (count unknown)	42 (1.1%)	29%	71%	
Unknown	114 (2.9%)	45%	55%	
Clinical M-category				0.85
cM0	3837 (97%)	31%	69%	
cM1	34 (0.9%)	29%	71%	
Unknown	99 (2.5%)	34%	66%	
Neoadjuvant therapy				0.05
No	324 (8.2%)	28%	73%	
Chemotherapy	253 (6.4%)	26%	74%	
Chemoradiotherapy	3373 (85%)	32%	68%	
Surgical approach				< 0.001
TTE thoracic part open	694 (18%)	27%	73%	
TTE thoracic part MI	1984 (50%)	18%	82%	
THE open	935 (24%)	56%	44%	
THE MI	344 (8.7%)	45%	55%	
Unknown	13 (0.3%)	69%	31%	
Salvage resection				0.75
No	3870 (98%)	31%	69%	
Yes	55 (1.4%)	29%	71%	
Unknown	45 (1.1%)	29%	71%	
Hospital volume (average number of resections/year)				< 0.001
0–25	522 (13%)	53%	47%	
26–50	2194 (55%)	29%	71%	
50 +	1229 (31%)	24%	76%	
Stopped before 2014	25 (0.6%)	76%	24%	
Year of resection				< 0.001
2011	491 (12%)	50%	50%	
2012	613 (15%)	39%	62%	
2013	641 (16%)	37%	63%	
2014	702 (18%)	26%	74%	
2015	778 (20%)	24%	76%	
2016	745 (19%)	20%	80%	

*ASA* American Society of Anesthesiologists, *TTE* transthoracic esophagectomy, *THE* transhiatal esophagectomy, *MI* minimally invasive, *LNs* lymph nodes

*Chi-squared analysis, in case of < 5% “unknown,” this category was not included in the statistical analysis (exception: cN-category)

**Table 2:** Multivariable logistic regression analysis for factors associated with ≥ 15 LNs

Characteristic	*n*	Multivariable analysis		
Total	3970	*P* value	OR	95% CI
Age (years)		0.29		
0–64	1756		ref	
65–74	1615	0.67	0.96	0.82–1.14
75 +	521	0.12	0.83	0.66–1.05
Preoparative weight loss (kg)		0.01		
0–10	2938		ref	
10.1–15	261	0.12	0.79	0.59–1.06
>15	174	0.19	0.78	0.54–1.13
Unknown	519	< 0.001	0.71	0.57–0.88
Tumor location in esophagus		0.59		
Cervical	4	0.41	0.40	0.05–3.46
Proximal	39	0.22	1.80	0.71–4.54
Mid	480	0.68	0.95	0.74–1.22
Distal	2451		ref	
Gastroesophageal junction	918	0.59	1.05	0.87–1.27
ASA score		0.77		
I–II	3020		ref	
III+	872		1.03	0.85–1.24
Charlson score		0.02		
0	1897		ref	
1	998	0.68	0.96	0.80–1.16
2 +	997	0.01	0.76	0.63–0.92
Clinical N-category		0.02		
cN0	1383		ref	
cN1	1553	0.37	1.08	0.91–1.29
cN2	707	0.02	1.32	1.05–1.65
cN3	99	0.15	1.47	0.87–2.48
cN+ (count unknown)	41	0.30	1.50	0.70–3.19
Unknown	109	0.07	0.67	0.43–1.03
Neoadjuvant therapy		< 0.001		
No	322	< 0.001	1.73	1.29–2.32
Chemotherapy	249	< 0.001	2.15	1.54–3.01
Chemoradiotherapy	3321		ref	
Surgical approach		< 0.001		
TTE thoracic part open (incl. MI abdomen)	686		ref	
TTE thoracic part MI	1968	0.004	1.38	1.11–1.73
THE open	912	< 0.001	0.29	0.23–0.36
THE MI	326	< 0.001	0.43	0.32–0.59
Hospital volume (average number of resections/year)		< 0.001		
0–25	506		ref	
26–50	2174	< 0.001	1.94	1.55–2.42
> 50	1212	< 0.001	3.01	2.36–3.83
Year of resection		< 0.001		
2011	462		ref	
2012	599	0.01	1.48	1.13–1.94
2013	616	0.00	1.53	1.17–2.00
2014	699	< 0.001	2.28	1.73–3.00
2015	774	< 0.001	2.44	1.85–3.21
2016	742	< 0.001	2.54	1.91–3.39

*ASA* American Society of Anesthesiologists, *TTE* transthoracic esophagectomy, *THE* transhiatal esophagectomy, *MI* minimally invasive, *OR* odds ratio, *CI* confidence interval

Factors associated with ≥ 15 LNs were clinical N2 category (reference: clinical N0, 1.32 [1.05–1.65]), no neoadjuvant therapy and neoadjuvant chemotherapy (reference neoadjuvant chemoradiotherapy, 1.73 [1.29–2.32] and 2.15 [1.54–3.01]), resection in a hospital with 26–50 or > 50 resections per year (reference: 0–25 resections, 1.94 [1.55–2.42] and 3.01 [2.36–3.83]), and resection between 2012 and 2016 (reference: 2011, ORs 1.48 [1.13–1.94], 1.53 [1.17–2.00], 2.28 [1.73–3.00], 2.44 [1.85–3.21], and 2.54 [1.91–3.39] for the years 2012 through 2016).

Transthoracic (open or minimally invasive) approach was associated with a higher percentage of patients with ≥ 15 LNs (versus open or minimally invasive transhiatal approach, 0.29 [0.23–0.36] and 0.43 [0.32–0.59]).

Stratified multivariable analysis for transthoracic resections showed a statistically significant association of minimally invasive approach with yield ≥ 15 LNs (reference: open transthoracic approach, 1.46 [1.15–1.85]). There was no such association for minimally invasive transhiatal resection with ≥ 15 LNs (reference: open transhiatal resection, 1.31 [0.97–1.75]).

### LN Yield in Relation to Short-term Surgical Outcomes

Table 3 presents the association of ≥ 15 LNs with short-term outcomes (with < 15 LNs as reference group). LN yield ≥ 15 was independently associated with fewer intraoperative complications (4.5% vs. 6.8%, OR 0.69 [0.50–0.95]). Postoperative complications were more frequent in patients with ≥15 LNs than in patients with < 15 LN, but multivariable analysis showed no statistically significant association (Table 3).

**Table 3:** Surgical outcomes associated with ≥ 15 LNs

Outcomes	< 15 LNs % (*n*)	≥15 LNs % (*n*)	Univariable analysis		Multivariable analysis	
			(with outcomes as dependent variable)			
			OR [95% CI] ≥ 15 LNs	*P* value	OR [95% CI] ≥ 15 LNs	*P* value
Positive resection margins	5.6% (68)	4.9% (132)	1.16 [0.86–1.57]	0.33		
Intraoperative complications	6.8% (83)	4.5% (122)	0.64 [0.48–0.86]	0.003	0.69 [0.50–0.95]^	0.02
Bleeding (with transfusion)	22% (18)	16% (20)				
Intestinal injury	9.6% (8)	5.8% (7)				
Spleen injury	13% (11)	17% (20)				
Other	55% (46)	61% (75)				
Postoperative complications	57% (702)	61% (1667)	1.17 [1.02–1.34]	0.02	1.01 [0.93–1.27]*	0.28
Pulmonary	29% (356)	32% (879)				
Cardiac	12% (150)	15% (401)				
Anastomotic leakage/local necrosis conduit	20% (241)	18% (503)				
Chylous leakage	5% (58)	8% (240)				
Severe postoperative complications	28% (339)	31% (847)	1.18 [1.01–1.37]	0.03	1.00 [0.85–1.19]*	0.98
30-day/in-hospital mortality	4.2% (52)	3.5% (95)	0.82 [0.58–1.15]	0.24		

*ASA* American Surgical Association, *LNs* lymph nodes, *OR* odds ratio, *CI* confidence interval

^Adjusted for: Body Mass Index, ASA score, surgical approach, year of resection

*Adjusted for: age, Body Mass Index, Charlson score, ASA score, histological type, tumor location, surgical approach, hospital volume

## Discussion

Between 2011 and 2016, the percentage of patients with at least 15 retrieved LNs in esophageal cancer surgery increased on a national level as well as for the individual hospitals.

Our results show an association of ≥ 15 LNs with higher clinical N-category. It may be possible that, in patients with clinically suspicious positive lymph nodes, the surgeon is particularly focused on more complete LN dissection. Also, tumor-positive LNs are often increased in size and therefore easier to identify during the operation and during pathological examination of the resection specimen. This could result in a higher number of retrieved LNs. Another explanation is that the immune response against the tumor influences the number of retrieved LNs. It has been suggested that larger tumors may cause a more intense immune response, leading to hyperplasia of local LNs, which could increase LN detectability.^10^ However, this hypothesis is not proven yet.

It is well known that the type of surgical approach in esophageal resection influences the number of retrieved LNs; i.e., transthoracic as compared with transhiatal approach is associated with a higher number of LNs retrieved, as also seen in the current study.^11^,^12^ Regarding the impact of a minimally invasive approach on LN yield, conflicting results have been published. A systematic review showed no differences between open and minimally invasive surgery, while another meta-analysis showed significantly higher LN retrieval in minimally invasive surgery (16 vs. 10, *P* = 0.03).^13^,^14^ In the present study, higher LN retrieval was seen especially in minimally invasive transthoracic procedures, which is in accordance with a recent propensity-score-matched analysis also using data from the DUCA [20 (2–59) vs. 18 (0–53) LNs; *P* < 0.001].^15^ It is possible that minimally invasive surgery offers benefits in terms of magnification and visibility of surgical structures and planes, which may translate into higher LN yield.

Busweiler et al. recently showed that, in patients undergoing gastrectomy, the percentage of patients with ≥ 15 retrieved LNs was higher in hospitals with higher composite hospital volume (gastrectomies, esophagectomies, and pancreatectomies).^16^ In our study, a similar association was noticed for esophageal cancer surgery. It is suggested that hospitals performing this type of surgery may benefit from the in-hospital experience.^16^ More intensive cooperation of a multidisciplinary team could be important for quality improvement initiatives.

This study showed an increase in the number of LNs every year. It is expected that, since the introduction of quality indicators in the DUCA, quality improvement initiatives in all hospitals have been initiated, because the results of these indicators are transparent for all individual hospitals each year. The national healthcare inspectorate, health insurance authorities, and different federations use the outcomes of this indicator to assess the quality of upper gastrointestinal surgical care in hospitals in The Netherlands. The increased numbers of retrieved LNs over the years could be the result of increased awareness of the importance of LN dissection by surgeons. On the other hand, back table dissection of the specimen and more extensive pathological assessment as a result of dedication of the pathologist could be major explanations as well. All these explanations have likely contributed to improving quality of care. The role of the pathologists in identifying nodes in the resection specimen is very important, as the time spent doing this makes a great difference.^17^ In this study, the role of the pathologist could not be studied, but dedicated pathologists or technicians are associated with increased number of nodes detected.^18^,^19^

More extensive LN dissection may lead to better locoregional tumor control. However, the importance of LN dissection for locoregional tumor control has been debated since the introduction of neoadjuvant chemoradiotherapy. It is known that neoadjuvant chemoradiotherapy leads to tumor and lymph node downstaging, resulting in more resections with negative margins and lymph nodes.^20^ The study of Talsma et al. showed that the number of retrieved LNs had a prognostic impact for patients who underwent surgery without neoadjuvant chemoradiotherapy, but not in the group of patients who underwent neoadjuvant chemoradiotherapy.^21^ For patients treated with neoadjuvant chemotherapy, Markar et al. also showed lower recurrence rate and improved survival for patients with higher lymph node yield. Similarly, effects of higher lymph node yield on survival or recurrence were not observed in patients treated with neoadjuvant chemoradiotherapy.^22^ In the current study, we observed an inverse correlation between neoadjuvant chemoradiotherapy and retrieved LNs, which has been reported before.^11^,^21^,^23^,^24^ An explanation for this phenomenon could be that use of neoadjuvant chemoradiotherapy leads to less priority for extended LN dissection by Dutch surgeons, or that neoadjuvant treatment, especially neoadjuvant chemoradiotherapy, may induce regression of LNs, as reported before.^10^ So, despite radical resection, fewer LNs are retrieved or detected by the pathologist. Unfortunately, the DUCA registry has no long-term follow-up. Hence, it cannot be concluded from the results of this study whether the number of retrieved LNs is a valid indicator for the *quality of locoregional tumor treatment*. Nonetheless, this indicator may be meaningful as an indicator for *overall quality of esophageal cancer care*. Higher number of retrieved LNs may lead to improved tumor staging, and complete pathological staging is essential to predict the prognosis of patients. Furthermore, in patients treated with neoadjuvant chemoradiotherapy, signs of tumor regression in LNs (instead of positive LNs) are a better predictor of prognosis than clinical N-category, which is not always easy to assess preoperatively.

## Conclusions

Pro and contra arguments can be provided for use of a minimal number of retrieved LNs as a quality indicator in clinical auditing. An argument for the use of this indicator in clinical auditing is that it reveals relevant variation in outcomes of hospitals, which seems to distinguish between them. Another advantage could be that this indicator may lead to better quality of esophageal cancer because of quality improvement initiatives. However, the validity of this indicator as a direct measure of the quality of LN dissection is questionable, and the effect of more retrieved LNs on tumor control is debatable since the introduction of neoadjuvant chemoradiotherapy. Nevertheless, higher lymph node retrieval does not seem to lead to higher morbidity or mortality, so the number of retrieved LNs can safely be used as an indicator for quality of care.

## Electronic Supplementary Material

Below is the link to the electronic supplementary material.

Supplementary material 1 (DOCX 46 kb)
