# Supplementary material for: A Population-based Study on Lymph Node Retrieval in Patients with Esophageal Cancer: Results from the Dutch Upper Gastrointestinal Cancer Audit
Source: Ann Surg Oncol. 2018 Mar 9;25(5):1211–20. doi: 10.1245/s10434-018-6396-7 (PMC5891559; doi:10.1245/s10434-018-6396-7)
Supplement: Supplementary file 1 — Supplementary material 1 (DOCX 46 kb) [file 10434_2018_6396_MOESM1_ESM.docx]

Patients registered in the DUCA between 2011 - 2016 with primary esophageal cancer who underwent an elective esophageal resection with curative intention. *n*=4076

Patients eligible to analyze

*n*= 3970

Excluded:

- 12 patients because their date of birth was unknown

- 80 patients because the status after 30 days was missing/unknown

- 14 patients because the number of lymph nodes was unknown

Supplementary figure 1. Flowchart for inclusion
